# Left Hemisphere Specialization for Oro-Facial Movements of Learned Vocal Signals by Captive Chimpanzees

**DOI:** 10.1371/journal.pone.0002529

**Published:** 2008-06-25

**Authors:** Elizabeth A. Reynolds Losin, Jamie L. Russell, Hani Freeman, Adrien Meguerditchian, William D. Hopkins

**Affiliations:** 1 Division of Psychobiology, Yerkes National Primate Research Center, Atlanta, Georgia, United States of America; 2 Department of Psychology, Agnes Scott College, Decatur, Georgia, United States of America; 3 Interdepartmental Program in Neuroscience, University of California Los Angeles, Los Angeles, California, United States of America; University of St. Andrews, United Kingdom

## Abstract

**Background:**

The left hemisphere of the human brain is dominant in the production of speech and signed language. Whether similar lateralization of function for communicative signal production is present in other primates remains a topic of considerable debate. In the current study, we examined whether oro-facial movements associated with the production of learned attention-getting sounds are differentially lateralized compared to facial expressions associated with the production of species-typical emotional vocalizations in chimpanzees.

**Methodology/ Principal Findings:**

Still images captured from digital video were used to quantify oro-facial asymmetries in the production of two attention-getting sounds and two species-typical vocalizations in a sample of captive chimpanzees. Comparisons of mouth asymmetries during production of these sounds revealed significant rightward biased asymmetries for the attention-getting sounds and significant leftward biased asymmetries for the species-typical sounds.

**Conclusions/Significance:**

These results suggest that the motor control of oro-facial movements associated with the production of learned sounds is lateralized to the left hemisphere in chimpanzees. Furthermore, the findings suggest that the antecedents for lateralization of human speech may have been present in the common ancestor of chimpanzees and humans ∼5 mya and are not unique to the human lineage.

## Introduction

Clinical and experimental evidence accumulated over the past 150 years has firmly established that the left hemisphere of the human brain is fundamentally involved in the perception and production of linguistic information [1], [2]. Left hemisphere dominance in linguistic functions is not restricted to specific modalities of communication and, to some extent, is modulated by the handedness of the individual [3]–[6]. Although historically hemispheric specialization has been considered a unique hallmark of human evolution, more recent studies in a host of vertebrates have provided evidence of population-level behavioral and brain asymmetries, suggesting that language is not a necessary condition for the expression of hemispheric specialization [7]–[9]. Notwithstanding, there remains intense scientific debate over whether animals, and particularly nonhuman primates, show hemispheric specialization in the perception and production of species-typical communicative signals and how this might relate to the evolution of language in humans [1], [8], [10]–[14].

With respect to perception of species-typical communicative signals, behavioral and neurological research has yielded somewhat inconsistent findings [15], [16]. For example, a number of investigators have examined behavioral responses or orienting asymmetries in response to species-typical sounds in rats, birds, sea lions, and nonhuman primates including vervet monkeys, rhesus macaques, Japanese macaques, barbary macaques and bonobos. Left hemisphere asymmetries have been reported in many species [17]–[24] while right hemisphere asymmetries have been reported in vervet monkeys [25]. In barbary macaques, no hemispheric asymmetry in response to species-typical calls was found [26]. Ablation studies and, more recently, functional imaging studies have similarly revealed mixed results [15], [16]. For example, in Japanese macaques, lesions to the posterior region of the left temporal lobe induced greater transient deficits in auditory discrimination of species-typical “coo” calls compared to right hemisphere lesions [27]. In contrast, in rhesus macaques, mainly right hemisphere biased asymmetries were found in cerebral glucose metabolism (measured by positron emission tomography (PET)) in response to species-typical vocalizations within the middle and posterior temporal lobe whereas a significant left hemisphere bias was found in the left temporal pole [28].

In contrast to studies on perception, far fewer studies on asymmetries in the production of species-typical sounds have been conducted. Most well known are the studies by Nottebohm and colleagues in song birds showing that lesions to the left but not right hypoglossal nerve result in significant deficits in song production (reviewed in Nottebohm [29]), though some bird species do show different directional biases in song production [30]–[32]. Leftward biases in sound production have also been reported in frogs [33]. In chimpanzees, population-level right-handedness has been reported for manual gestures [34]. Similar evidence of right-handedness for species-typical gestures has been reported in gorillas [35] and baboons [36].

One behavioral manifestation of the asymmetries in the cortical control of human speech production is the lateralization of oro-facial movements. Specifically, the right side of the mouth moves first and is more expressive when producing words whereas the left half of the mouth is more animated during emotional expression [37]–[40]. Evidence of oro-facial asymmetries in relation to speech production has also been reported in infants, suggesting that left hemisphere asymmetries in linguistically-relevant vocal production are present early in life [41]. Studies of asymmetries in facial expressions associated with the production of species-typical vocalizations in nonhuman primates including marmosets, rhesus monkeys, and chimpanzees have largely reported right hemisphere biased asymmetries, suggesting that, if nonhuman primates follow the human pattern of hemispheric specialization for the production of these types of signals, the sounds and associated facial expressions studied in these species are indicative of emotional valence rather than linguistic or referential information [42]–[45].

Recent studies, however, have demonstrated that captive chimpanzees can learn to voluntarily produce novel sounds to capture the attention of an otherwise inattentive human [46]–[51]. Two such sounds described in chimpanzees are the “raspberry” and “extended grunt” [52]. The raspberry sound (known elsewhere as the ‘splutter’ or the ‘Bronx cheer’) is an unvoiced bilabial trill with a velaric egressive airstream mechanism in which the chimpanzees purse their lips and expel air out from their mouths rather than their lungs, vibrating their lips together to produce sound. van Schaik et al. [53] and, more recently, Cartmill and Byrne [54] have reported that a sound similar to the raspberry is produced in some populations of wild and captive orangutans. The extended grunt is a low frequency but noisy voiced sound that the chimpanzees make with their mouths open while expelling air from the lungs. Although the raspberry has not been described in wild chimpanzees, there is some evidence that the extended grunt has been recorded in wild chimpanzees, at least those at Gombe [55] (p. 131), though only a verbal description has been provided and no spectrogram, so it is not clear if the exact same sound is being produced in wild and captive chimpanzees.

With respect to the function of the raspberry sound and the extended grunt vocalization, several reports have demonstrated that chimpanzees will selectively produce these sounds, and not species-typical food calls, in greater frequency when both food and a human are in proximity to the chimpanzees compared to when only food or only a human are present. In addition, chimpanzees made more species-typical food vocalizations when only food was present but not when only a human was present, or when both food and human were present [46]. These results suggest that chimpanzees are choosing to use the raspberry and extended grunt sounds depending on the presence or absence of a human in conjunction with food. Captive chimpanzees have also been reported to use the raspberry and extended grunt more frequently when a) a human is facing away from them compared to towards them or b) when a human is offering food and looking at a chimpanzee living in the same cage as the focal subject compared to when the human is offering food and looking directly at the focal subject [50]. In contrast to the attention-getting use of the raspberry and extended grunt in captivity, a sound similar to the extended grunt measured in our study is produced by chimpanzees living at Gombe during nesting behavior [55]. Thus, whether the raspberry or extended grunt are observed in wild chimpanzees and other apes remains a topic of interest and continued research. Notwithstanding, it does appear that the functional *use* of both the raspberry and the extended grunt as an attention-getting mechanism is unique to chimpanzees living in captive environs, suggesting that the chimpanzees have learned to use these sounds in a novel social-cognitive setting.

The differential use of the raspberry and extended grunt in response to orienting or attentional cues of humans, further suggests that these sounds are referential and produced intentionally, in contrast to the majority of primate vocalizations which are widely believed to consist of emotional information and not to be intentionally produced. Because the raspberry is an arbitrary sound, and because both the raspberry and extended grunt have been acquired and are used in different contexts than species-typical calls of chimpanzees [52], in the current study we examined whether facial expressions associated with the production of these sounds were differentially lateralized compared to facial expressions associated with the production of species-typical vocalizations.

For comparison to the raspberry and extended grunt sounds, oro-facial asymmetries associated with two species-typical vocalizations, food-barks and pant-hoots, were measured. These are two vocalizations produced in different emotional contexts by chimpanzees. Food–barks are repeated and often high-pitched barks produced by expelling air from the lungs with the lips slightly parted and mouth corners withdrawn. Food-barks are produced when arriving at food sites or when ingesting highly preferred foods. Pant-hoots are repeated voiced calls consisting of alternating “hoo” vocalizations, produced with forward-protruding rounded lips, and voiced inhalations, during which the mouth is open wide. Pant-hoots are distance calls and are used in several behavioral contexts including when arriving at food sites, when greeting familiar individuals, and during bluff displays [55]. The vast majority of pant-hoots observed in this study were produced during displays directed towards humans or other chimpanzees, a negatively-valenced emotional context. The production of food-barks and pant-hoots in these emotional contexts has been reported in a number of chimpanzee populations both in the wild and in captivity and thus these vocalizations appear to be neither functionally used nor learned in the same way as the raspberry and extended grunt [55], [56].

The oro-facial asymmetry of pant-hoot vocalizations was measured previously by Fernández-Carriba et al. [42] and therefore inclusion of this class of sounds was largely for the purpose of replication of the method. Food-bark vocalizations have not been previously studied but are of interest because they are a positive emotional expression in chimpanzees. Davidson [57] has suggested that positive and negative emotions are differentially produced by the left and right hemispheres, with positive emotions being controlled by the left hemisphere. Fernández-Carriba et al. [42] failed to find evidence of a rightward oro-facial bias for positively-valenced play faces, as would have been predicted by Davidson's hypothesis, but instead found that that play faces were left biased as were negatively-valenced expressions such as the silent bared-teeth and scream face. It is important to note that the play faces that were evaluated by Fernández-Carriba et al. [42] were primarily produced by younger subjects. Thus, including food-barks in this study allowed us to further evaluate the potential role of emotional valence on oro-facial asymmetries in adult chimpanzees. Based on the human pattern of hemispheric dominance in the production of learned, referential signals versus emotional signals, and the failure of Fernández-Carriba et al. [42] to find differential oro-facial lateralization based on emotional valence, we predicted that the raspberry and extended grunt would exhibit a right oro-facial bias suggesting left hemisphere dominant control while the pant-hoot and food-bark would exhibit a left-oro-facial bias suggesting right hemisphere dominant control.

## Methods

### Subjects

Digital video images were collected in a sample of captive chimpanzees (*Pan troglodytes*) (n = 69) housed at the Yerkes National Primate Research Center (YNPRC) while they produced four different types of sounds: pant-hoots, food-barks, raspberries, and extended grunts (see Table 1 for number of individuals producing each expression type and number of images analyzed and rejected). Sample sizes varied across sound types because we could not control which animals produced sounds during any given video collection time period. The subjects ranged in age from 5–44 years (*Mean* = 18.69, s.d. = 8.72).

**Table 1:** Summary of Expression Analyses.

Expression Type	#Individuals	#Exemplars Analyzed	#Exemplars Rejected
Rater 1 Original Measurements			
Pant-hoot	32	68	57
Food-bark	32	73	46
Raspberry	35	100	127
Extended grunt	10	26	28
Total	69*	267	258
Rater 1 and Rater 2 Reliability Measurements			
Pant-hoot	23	32	
Food-bark	22	35	
Raspberry	29	46	
Extended grunt	7	15	
Total	53*	128	

*Note:* We analyzed a maximum of 3 expressions from an individual in an expression category so not all rejected exemplars are poor quality images. ^*^ The total number of individuals is less than the sum of individuals contributing to each expression category because some of the same animals contributed to multiple expression categories.

### Materials and Apparatus

Video was recorded in NTSC format (30 f/sec) with Cannon ZR 20, ZR 70 and ZR 90 digital video cameras on miniDV tapes and transferred onto a Dell Dimension 4550 computer using Roxio Videowave Movie Creator version 1.6.676.1 for further analysis. This program was also used to capture still images from video sequences. Adobe Photoshop version 6.0 and Scion Image beta 4.0.2 were used to manipulate and make measurements on the still images. When food was used to elicit expressions, we typically used bananas and apples. Occasionally grapes, grapefruit, and frozen Kool-aid were also used, depending on availability and the individual preferences of the focal subjects.

### Procedure

Behavioral sequences of the four facial expressions under study were filmed over a period of one year from April 2004–April 2005. Video recordings of pant-hoot expressions were made ad libitum in the context of the normal social interactions of the chimpanzees. Usable still images of pant-hoot expressions were captured primarily during display behavior directed towards humans because it was most likely that chimpanzees would be facing the experimenter in this context (see [55]). The food-bark, raspberry and extended grunt were filmed during the presentation of a food item by a research assistant. Expressions were filmed during two-hour blocks of time, one in the morning and one in the afternoon, three days a week, resulting in a total of approximately 120 hours of video for analysis.

Still images of expressions were selected for oro-facial asymmetry analysis using a three-step process. First, all videotaped facial expression sequences were viewed by the primary author in a frame-by-frame manner, and the frame depicting the point of greatest exaggeration of the expression was isolated. During production of the food-bark, pant-hoot (“hoo” component), and extended grunt expressions, this point occurred when the mouth was open the widest. During production of the raspberry, this point occurred when the bottom lip was farthest extended and sound was produced. Second, consistent with previous studies in chimpanzees and other nonhuman primates [43], [44], [55] the following criteria were used to determine whether the isolated frame would be captured for analysis: 1) the frame must have been in focus, 2) the chimpanzee's face must have been completely frontal to the camera based on visual assessment, and 3) when expressions occurred in bouts, the clearest and most frontal of the expressions in a bout was selected. The third selection step occurred after captured images were imported into Adobe Photoshop, blown up, and inspected closely. At this point, two additional criteria were used to select images for oro-facial asymmetry analysis from the still frames captured in step two: 1) since much of the video used in this study was filmed through the wire mesh of the chimpanzees' enclosures, which could obscure facial landmarks needed for analysis, all facial landmarks, such as eye corners, must have been visible or their position must have been easily inferred, 2) a second check for lack of facial rotation was performed. Shown in Table 1 are the total number of still images analyzed and rejected based on these criteria (both analyzed and rejected images can be obtained from the corresponding author). For most expressions (63%) included in the analysis, three images of that expression were analyzed. We have also included data for expressions of which we only have one (19%) or two (17%) useable images from an individual in order to increase the sample size of individuals and expressions in our study.

### Image Analysis

**Figure 1:**
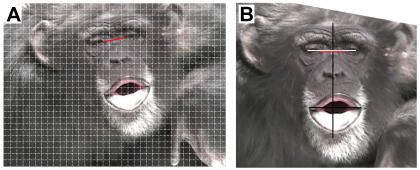
Examples of image analysis procedures. (a) Rotation of the face into vertical position using grid and line between inner eye corners in Adobe Photoshop. (b) Bisection of the face through midpoint of line connecting inner eye corners and additional lines drawn between midline and outer eye and mouth corners.

All images were analyzed using the measurement procedure pioneered by Hook-Costigan and Rogers in marmosets [44] and later used by Fernández-Carriba et al. [42] in chimpanzees (see Figure 1a & b). In this procedure, a line was drawn between the inner corners of the eyes and compared to the horizontal lines on a fixed grid in Adobe Photoshop in order to rotate the face into a vertical position (See Figure 1a). A line was then drawn between the outer corners of the eyes (see Figure 1b). The image was then saved in TIFF format and imported into the Scion Image software. Pixel distance or pixel area was calculated in Scion Image by using the cursor to trace a line or outline an area of an image for measurement. In Scion Image, the midpoint of the line between the inner eye corners was calculated and a perpendicular line was drawn at this point to bisect the face (see Figure 1b). Lines were also drawn from the outer corners of the mouth to the midline.

**Figure 2:**
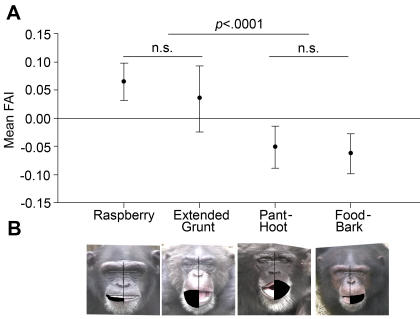
Learned (raspberry and extended grunt) and species-typical expressions (pant-hoot and food-bark) are differentially lateralized. (a) Least squares means of FAI scores for the raspberry, extended grunt, pant-hoot and food-bark expressions along with 95% confidence intervals for these values. Positive FAI scores represent right hemi-mouth biases and negative values reflect left hemi-mouth biases. (b) Illustration of hemi-mouth area calculation procedure on representative images of the raspberry, extended grunt, pant-hoot and food-bark under their corresponding mean FAI values.

Once all the lines were drawn, the pixel distances between the outer eye corners and mouth corners to the midline were calculated. The left and right hemi-mouth pixel areas were also measured for each expression by tracing around the outer border of the lips to the midline on each side of the mouth (See Figure 2b for examples of mouth-tracings for area measurements of each expression type). Facial asymmetry indices (FAIs) were calculated for the distances to the outer eye corners (eye FAIs) and the mouth area (mouth area FAIs) by subtracting the left from the right side and dividing that value by the sum of the right side and left side measurements. Negative FAI values indicate leftward biases and positive FAI values indicate rightward biases.

The mouth area FAIs were adjusted for possible asymmetries in the image due to rotation of the face relative to the camera using the previously established method of subtracting the eye FAIs of each image from the mouth area FAI values for that image [42], [44]. These adjusted FAI values for mouth area were then used in the remaining analyses. It is important to note that analyzed images contained very little facial rotation; Student's t tests revealed that the eye FAI values did not differ significantly from zero in any expression category and subtraction of these values from the mouth area FAIs did not significantly impact our findings.

To assure reliability in the measurement of the areas of the left and right halves of the mouth, a second experimenter (rater 2), naive to the hypothesis of this study, performed the measurement procedure outlined above on randomly-selected images totaling approximately half of the images originally assessed by rater 1 (n = 128) from each of the 4 expression categories (see Table 1 for number of images and individuals contributing to reliability sample). To prevent rater-introduced bias, a random sample of images from each expression category was flipped on the left-right axis, prior to re-measurement by rater 2. A Pearson Product Moment correlation coefficient of the FAI measurements between raters 1 and 2 was positive and significant (*r* = .725, df = 126, *p*<.01), suggesting good agreement between the raters. As an additional measure of inter-rater reliability, which is also sensitive to systematic bias, we calculated an intraclass correlation coefficient (ICC) using a two-way mixed effects model for absolute agreement of single measurements. The resulting ICC was .724 and no systematic differences between raters were detected (*F*_(1,127)_ = .381, *p* = .538), again indicating good agreement between raters 1 and 2.

For our statistical analysis we used SAS to perform a mixed model ANOVA with nesting on the complete data set measured by rater 1. In the model, expression type (n = 4) was a fixed factor and subject (n = 69) was a random factor within which the individual exemplars of each expression were nested. For the overall analyses, the FAI measures from rater 1 served as the dependent variable.

## Results

### Asymmetry in Oro-Facial Expressions

We separately incorporated sex as a fixed factor, and age as a covariate into our model and the effects of neither variable were significant, so these factors were dropped from the model for further analysis. Mean mouth area FAI values were significantly negative (leftward) for the pant-hoot (*t*(90) = −2.74, *p* = .0073) and food-bark (*t*(90) = −3.43, *p* = .0009), and significantly positive (rightward) for the raspberry (*t*(90) = 3.82, *p* = .0002) (Figure 2a). The mean mouth area FAI value for the extended grunt did not differ significantly from zero, though the mean was positive like the raspberry, and we had a relatively small sample (26 expressions from10 individuals) that produced this expression (Figure 2a).

The mixed model ANOVA revealed a significant main effect of expression on mouth area FAIs (*F*_(3,90)_ = 14.74, *p*<.0001). A post-hoc main comparison between the two learned expressions (raspberry and extended grunt) and the two species-typical emotional expressions (pant-hoot and food-bark) revealed that learned expressions had significantly higher (more rightward) mouth area FAIs than those of the species-typical emotional expressions (*F*_(1,90)_ = 28.87, *p*<.0001).

Post-hoc pairwise comparisons (with Bonferroni correction) between the 4 expressions revealed that mouth area FAIs of the raspberry were significantly higher (more rightward) than those of the food-bark (*t*(90) = −5.9, *p*<.0001) and the pant-hoot (*t*(90) = −5.25, *p*<.0001). Additionally, mouth area FAIs for the extended grunt were significantly higher (more rightward) than those of the food-bark (*t*(90) = −2.99, *p* = .0214) and were borderline significantly higher than the pant-hoot (*t*(90) = 2.62, *p* = .0611).

**Figure 3:**
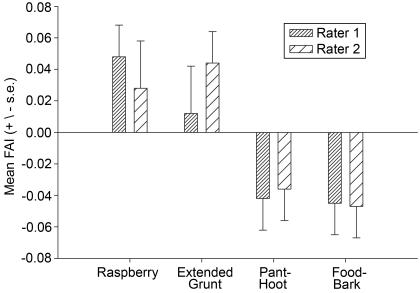
Inter-rater comparison. Mean FAI scores (+/− s.e.) for the raspberry, extended grunt, pant-hoot and food-bark expressions for raters 1 and 2. See Table 1 for sample sizes for each expression type.

Recall that for the purposes of reliability a second rater (rater 2) quantified the FAI for nearly half of the sample of images. As an additional means of evaluating consistency between the two raters, the mixed model ANOVA used on the original data set was carried out on the mouth area FAI measurements for the subset of images that were analyzed by both raters. Mean FAI values for both rater 1 and rater 2 were positive (rightward) for raspberry and extended grunt expressions and negative (leftward) for pant-hoot and food-bark expressions (Figure 3). There were significant main effects of expression type for both rater 1 (*F*_(3,38)_ = 4.39, *p* = .0096) and rater 2 (*F*_(3,38)_ = 3.31, *p*<.0302) and the learned expressions (raspberry and extended grunt) were significantly more rightward compared to the species-typical emotional expressions (pant-hoot and food-bark) for both rater 1 (*F*_(1,38)_ = 7.43, *p*<.0097) and rater 2 (*F*_(1,90)_ = 8.74, *p*<.0053). Thus, the same trends were seen in the measurements by the blinded rater (rater 2) as in the original data set (rater 1).

## Discussion

Our findings suggest that chimpanzee facial expressions are differentially expressed on the left and right sides of the face depending on the function of the sounds associated with each expression. Facial expressions associated with the production of raspberry sounds are expressed more intensely on the right side of the face suggesting that the left hemisphere is dominant in the motor control of these novel oro-facial movements. These results are similar to recent findings documenting population-level right-handedness for referential, manual gestures in chimpanzees [34] and more generally suggest that the left hemisphere might control the production of intentionally-produced communicative signals from at least two different sensory modalities in chimpanzees. In contrast, facial expressions associated with involuntary, unlearned, species-typical expressions, including pant-hoots and food-barks, are expressed more intensely on the left side of the face, consistent with the view that they reflect emotional expressions controlled by the right hemisphere.

As an alternative explanation of our results, the observed dichotomy in oro-facial asymmetries may reflect inherent differences between the left and right hemisphere as they relate to motor learning. Clinical and experimental studies suggest that motor movements, particularly complex movements made by the left and right hands, are under the control of the left premotor and supplementary motor areas [58], [59], [60]. Studies also indicate that the left hemisphere, compared to the right, is superior in motor skill acquisition and performance [61]. The raspberry sounds and extended grunt vocalizations used by the chimpanzees in this study are novel and have been acquired through inadvertent instrumental conditioning associated with the prolonged captivity experienced by these animals. The use of these novel, attention-getting sounds and facial expressions and their asymmetric expression on the right side of the face therefore might simply reflect the left hemisphere's superior motor acquisition ability [62] compared to the right, rather than reflect differences in the communicative capacities of the two hemispheres in chimpanzees.

Lastly, it might be suggested that because the raspberry is used in a social context, the right hemi-mouth bias reflects the positive valence effect of the left hemisphere, as has been reported for the “twitter” vocalization in marmosets [44]. We do not favor this explanation for our findings because other facial expressions associated with positive emotional expressions, such as the food-bark from this study and the play face from the study by Fernández-Carriba et al. [42], were found to be expressed more intensely on the left half of the face.

It should be emphasized that we are not suggesting that the chimpanzees have acquired volitional control of their vocalizations per se. The issue of whether nonhuman primates have volitional control of their vocalizations remains a topic of considerable debate [63], [64], [65] and these data do not speak directly to this issue. Recall that the raspberry sound is not a voiced signal but rather it is a sound made by the chimpanzees by expelling air through their lips. Thus, based on these data, we are suggesting that the chimpanzees can learn to manipulate their facial musculature to produce sounds that can be used in specific communicative contexts and that the left hemisphere is dominant in controlling the production of sounds acquired in this manner. From this perspective, it might be further argued that voluntary control of facial expressions may have preceded the evolution of volitional control of the vocal cords and other peripheral structures involved in the production of voiced signals [66], [67], [68], [69].

The presence of left hemisphere specialization in oro-facial motor control in the common ancestor of chimpanzees and humans may have set the stage for the evolution of more sophisticated motor systems including those innervating the tongue and vocal folds that allowed for the emergence of human speech. Arguably one of the initial and important requisite conditions for the emergence of spoken language had to be the ability to learn new sounds and to produce those sounds in functionally-meaningful contexts [63]. Our results suggest that these pivotal abilities may have been present in the common ancestor of humans and chimpanzees.
